# Full 10-20 EEG application in hospitalised neonates is not associated with an increase in stress hormone levels

**DOI:** 10.1016/j.cnp.2017.12.002

**Published:** 2017-12-19

**Authors:** Kimberley Whitehead, Laura Jones, Pureza Laudiano Dray, Judith Meek, Lorenzo Fabrizi

**Affiliations:** a Department of Neuroscience, Physiology and Pharmacology, University College London, London WC1E 6BT, United Kingdom; b Elizabeth Garrett Anderson Obstetric Wing, University College London Hospitals, London WC1E 6BD, United Kingdom

Neonatal EEG monitoring in pre-term and full-term infants offers a window onto neurological functioning, and clinically relevant insights into normative and aberrant development of cortical activity patterns (Whitehead et al., 2017). We have recently shown the importance of full 10/20 electrode coverage in neonatal EEG recordings, due to the prevalence of cortical bursting over the posterior temporal regions (electrodes T6/T5) (Whitehead et al., 2017). Nevertheless, 10/20 EEG application involves a relatively high level of handling and could be a potential stressor, especially in those infants who are already exhibiting higher stress levels.

Salivary cortisol reflects activity of the hypothalamic pituitary axis: increased cortisol concentration indicates increased stress. To assess the impact of EEG set-up on systemic stress levels in hospitalised infants, we measured salivary cortisol immediately before EEG application, and approximately 15 min after the completion of EEG application (median time between swabs: 46 min), based on the latency and duration of stressor-induced changes in cortisol concentration in infants (Ramsay and Lewis, 2003). EEG application occurred between 09:00 and 17:30. Subjects included 33 un-sedated infants with gestational age 26 + 4–41 + 6 weeks + days (median 36 + 4), corrected age 32 + 3–47 + 6 weeks + days (median 37 + 5), and postnatal age 0.5–95 days (median 5 days). Ethical approval was obtained from the NHS Health Research Authority and informed written parental consent was obtained prior to each study. Separate written consent was obtained to publish a photograph of an infant having electrodes placed.

Disposable Ag/AgCl cup electrodes were applied by an experienced clinical scientist (KW). The median number of electrodes applied was 18 (maximum 19), in addition to the ground and reference, single lead ECG and a respiratory monitor. Target impedance was <10 kΩ. All infants were offered individualised, developmentally appropriate comfort measures during EEG application as and when required (e.g. swaddling if they became unsettled). Saliva samples were collected pre- and post-EEG application by a research nurse (PLD) using a cotton swab. The swabs were then frozen at −20 °C until ready for analysis. Samples were assayed in duplicate when possible at a Salimetrics lab, using an enzyme immunoassay that has a lower limit of sensitivity of 0.007 µg/dL and a standard curve range from 0.012 to 3.0 µg/dL. The average intra- and inter-assay coefficients of variation were low (3.4% and 7.6%, respectively). Cortisol concentration pre- and post-EEG application was compared with paired Wilcoxon tests because they were not normally distributed (Shapiro-Wilk test). Unpaired variables were compared with Mann-Whitney or chi-squared tests. Data analysis was carried out using IBM SPSS version 22. Statistical significance was set at 0.05.

Median cortisol concentration pre-EEG application was 0.27 µg/dL (range 0.09–2.91), and post-EEG application was 0.37 µg/dL (range 0.03–1.74), values similar to those found in previous neonatal studies (Mörelius et al. 2016). There was no difference between cortisol concentration pre- and post-EEG application across the 33 infants (p = .201). In particular, this was also the case for vulnerable sub-groups (corrected age: pre-term (<37 weeks), n = 13; ward location: special care or high dependency unit, n = 14) (p ≥ 0.432).

Next, we investigated whether baseline stress level influenced the effect of EEG application by splitting infants into those with higher pre-EEG cortisol concentration (≥0.25 µg/dL, n = 17) and lower pre-EEG cortisol concentration (<0.25 µg/dL, n = 16). Infants with a higher pre-EEG cortisol value had a higher corrected age than infants with a lower pre-EEG cortisol value (p = .017), while there was no difference in sex distribution (p = .881), ward location (special care or high dependency unit vs. postnatal ward, p = .881), or mode of delivery (vaginal vs. caesarean section, p = .866). EEG application was associated with a decrease in cortisol concentration in infants who had a higher concentration at baseline (p = .023; median 0.31 µg/dL decrease, which is a median 41% decrease in the pre-EEG value), and no change in infants who had a lower cortisol concentration at baseline (p = .120) (Fig. 1).

**Fig. 1:**
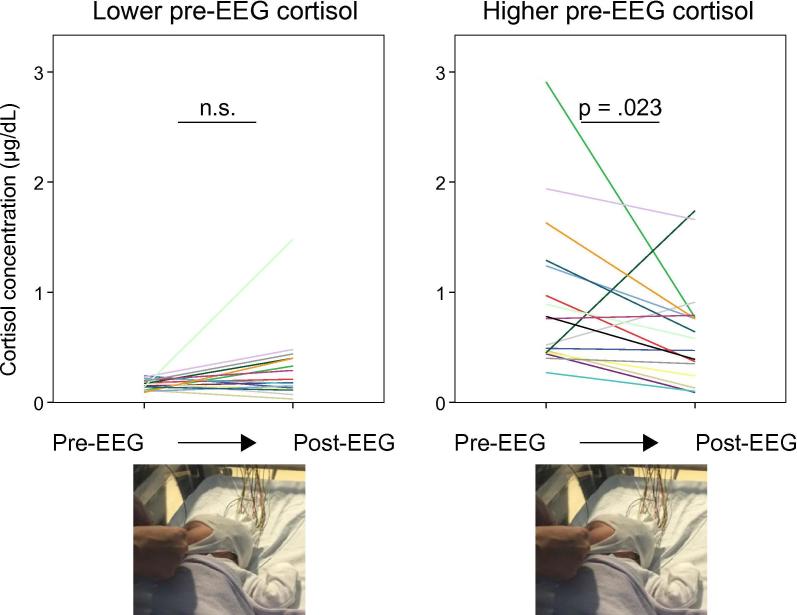
EEG application is not associated with any change in cortisol concentration in infants with a lower stress level at baseline, while it is associated with a decrease in cortisol concentration in infants with a higher stress level at baseline. Each coloured line represents one infant.

Stress in hospitalised neonates is associated with adverse clinical signs, including increased nociceptive cortical response following a necessary heel lance, and fluctuations in intracranial blood pressure (Jones et al., 2017, Mörelius et al., 2016). Consequently, it is important to assess the impact of interventions, like EEG application, on systemic stress levels. In this study we have shown that EEG application, alongside appropriate comfort measures, does not necessarily increase stress levels in hospitalised infants. Our results should be interpreted conservatively because the hospital environment and cumulative exposure to stressful procedures could mask or alter reactivity. Nevertheless, previous studies have demonstrated that salivary cortisol *can* significantly increase in hospitalised neonates following a non-invasive stressor lasting approximately as long as EEG application (neurological examination) (Mörelius et al., 2016). Therefore our finding that EEG application is not associated with an increase in stress levels is reassuring and supports our clinical impression (based on behavioural observation, and review of heart rate and pulse oximetry acquired by cot-side monitors where available) that EEG application can be well tolerated by both pre-term and full-term infants.

Interestingly we show that EEG application is actually associated with *decreased* stress levels in infants with higher stress levels at baseline. In line with this EEG-associated decrease in cortisol levels, tactile stimulation of the head and body decreases stress behaviours in neonates (Hernandez-Reif et al., 2007). Further, gentle time-limited sensory stimulation significantly reduces salivary cortisol concentration in neonates (music: Schwilling et al., 2015) and older infants (swimming; riding in car, irrespective of whether sleep occurs: reviewed in Jansen et al. 2010). Therefore it is possible that EEG application has the potential to be soothing when carried out by experienced staff, especially in conjunction with comfort measures as necessary, which neonates with higher stress levels may be more likely to both require and benefit from.

## Declarations of interest

None.
